# Lentinan Supplementation Protects the Gut–Liver Axis and Prevents Steatohepatitis: The Role of Gut Microbiota Involved

**DOI:** 10.3389/fnut.2021.803691

**Published:** 2022-01-20

**Authors:** Xiaoying Yang, Mingxuan Zheng, Menglu Zhou, Limian Zhou, Xing Ge, Ning Pang, Hongchun Li, Xiangyang Li, Mengdi Li, Jun Zhang, Xu-Feng Huang, Kuiyang Zheng, Yinghua Yu

**Affiliations:** ^1^Jiangsu Key Laboratory of Immunity and Metabolism, Department of Pathogen Biology and Immunology, Xuzhou Medical University, Xuzhou, China; ^2^Tianjin Third Central Hospital, Tianjin, China; ^3^Medical Technology Institute, Xuzhou Medical University, Xuzhou, China; ^4^Department of Laboratory Medicine, Affiliated Hospital of Xuzhou Medical University, Xuzhou, China; ^5^Affiliated Hospital of Liaoning University of Traditional Chinese Medicine, Shenyang, China; ^6^School of Medicine, Illawarra Health and Medical Research Institute (IHMRI), University of Wollongong, Wollongong, NSW, Australia

**Keywords:** mushroom, NAFLD, gut-liver axis, oxidative stress, gut microbiota, β-glucan

## Abstract

The microbiota–gut–liver axis has emerged as an important player in developing nonalcoholic steatohepatitis (NASH), a type of nonalcoholic fatty liver disease (NAFLD). Higher mushroom intake is negatively associated with the prevalence of NAFLD. This study examined whether lentinan, an active ingredient in mushrooms, could improve NAFLD and gut microbiota dysbiosis in NAFLD mice induced by a high-fat (HF) diet. Dietary lentinan supplementation for 15 weeks significantly improved gut microbiota dysbiosis in HF mice, evidenced by increased the abundance of phylum Actinobacteria and decreased phylum Proteobacteria and Epsilonbacteraeota. Moreover, lentinan improved intestinal barrier integrity and characterized by enhancing intestinal tight junction proteins, restoring intestinal redox balance, and reducing serum lipopolysaccharide (LPS). In the liver, lentinan attenuated HF diet-induced steatohepatitis, alteration of inflammation–insulin (NFκB-PTP1B-Akt-GSK3β) signaling molecules, and dysregulation of metabolism and immune response genes. Importantly, the antihepatic inflammation effects of lentinan were associated with improved gut microbiota dysbiosis in the treated animals, since the Spearman's correlation analysis showed that hepatic LPS-binding protein and receptor (Lbp and Tlr4) and pro- and antiinflammatory cytokine expression were significantly correlated with the abundance of gut microbiota of phylum Proteobacteria, Epsilonbacteraeota and Actinobacteria. Therefore, lentinan supplementation may be used to mitigate NAFLD by modulating the microbiota–gut–liver axis.

## Introduction

Nonalcoholic fatty liver disease (NAFLD), one of the most common liver diseases worldwide, affects up to 30% of the adult population (1). Nonalcoholic steatohepatitis (NASH), a more severe form of NAFLD, is defined by the presence of steatosis with inflammation and progressive fibrosis, ultimately leading to cirrhosis or hepatocellular carcinoma (2). Recently, evidence for cross talk among the gut microbiota, the liver, the immune system, and energy metabolism has emerged, indicating that the microbiota is an important player in the development of NASH (3).

Compelling evidence links NASH with the alteration of the gut microbiome and intestinal barrier integrity. For example, the diversity and composition of gut microbiota significantly alter in the animal model and patients with NASH (4, 5). The tight junction proteins, including zonula occludens-1 (ZO-1) and occludin, decrease in the proximal small intestine of NASH mice (6). The gut integrity is disrupted by a redox imbalance in the intestine (7–10). Reactive oxygen species (ROS), such as nitric oxide (NO) and its generating enzyme inducible NO synthase (iNOS), induce the dysfunction and loss of tight junction of intestinal epithelial cells (7–9). In contrast, antioxidant enzymes, such as haem oxygenase 1 (HO-1), NAD(P)H quinone dehydrogenase 1 (NQO1), and glutamate-cysteine ligase catalytic subunit (Gclc), play important roles in maintaining intestinal barrier integrity (10). The transcription factor of these antioxidant enzymes, nuclear factor erythroid 2-related factor 2 (Nrf2), ameliorates intestinal barrier dysfunction induced by lipopolysaccharide (LPS, present in the outer membrane of gram-negative bacteria) (11). Therefore, gut microbiota dysbiosis and redox imbalance may disrupt gut integrity and lead to overtranslating the gut bacteria-derived toxin, such as LPS, into the port vein, thereby promoting inflammatory responses in the liver.

Lipopolysaccharide and its downstream pathway significantly contribute to hepatic inflammation in NAFLD (12, 13). LPS binds to its binding protein (Lbp), a 60-kDa acute-phase protein, to elicit immune responses by presenting the LPS to toll-like receptor 4 (Tlr4, important cell surface pattern receptor) (14, 15). Activation of Tlr4 subsequently induces proinflammatory responses, including the activation of nuclear factor kappa B (NFκB) and the increase in proinflammatory cytokines (TNFα, IL-6, and IL-1β) expression (14, 15). NFκB is also a transcription factor that increases protein tyrosine phosphatase 1B (PTP1B, encoded by the ptpn1 gene) (16), leading to insulin resistance. It is reported that patients with NASH have upregulation of hepatic Tlr4 mRNA and increased serum LPS (hyperendotoxinemia) (17). Therefore, the alteration of the gut–liver axis mediated by gut microbiota metabolite, LPS, may play a critical role in the development and progression of NASH.

Mushrooms have been used as food and medicinal resources for millennia (18). A clinical study has reported that higher mushroom intake was negatively associated with the NAFLD prevalence among adults (19). It is speculative that the active ingredient in mushrooms, such as β-glucan, contributes to the beneficial effect. Lentinan from shiitake mushroom, a β(1,3)/β(1,6)-glucan, has a β(1,3) backbone branched with short β(1,6)-linked side chains. In comparison, the β-glucans in cereal are primarily in β(1,4) linkages separating shorter stretches of β(1,3) structure. The mixed-linkage β(1,3)/β(1,4)-glucan (MLG) of cereal is the specific hydrolysis by the MLG utilization locus (MLGUL) of some gut microbiota, such as *Bacteroides ovatus* (20), resulting in compositional and functional shifts in the gut microbiota (21, 22). Recently, it has been reported that lentinan, β(1,3)/β(1,6)-glucans of mushroom, alleviates LPS-induced intestinal injury through modulating the composition and metabolites of intestinal microbiota in piglet (23). However, lentinan's potential effects and mechanism on the gut–liver axis in NAFLD represent a gap in the current research.

In this study, using the high-fat (HF) diet-induced NASH mouse model, we examined the effects of lentinan on gut microbiota, gut redox balance, and tight junction proteins, and also lipid deposition, NFκB/PTP1B signaling of endotoxin LPS, macrophage infiltration, and transcriptome regulation of immune and metabolic response in the liver.

## Materials and Methods

### Materials

**Figure 1:**
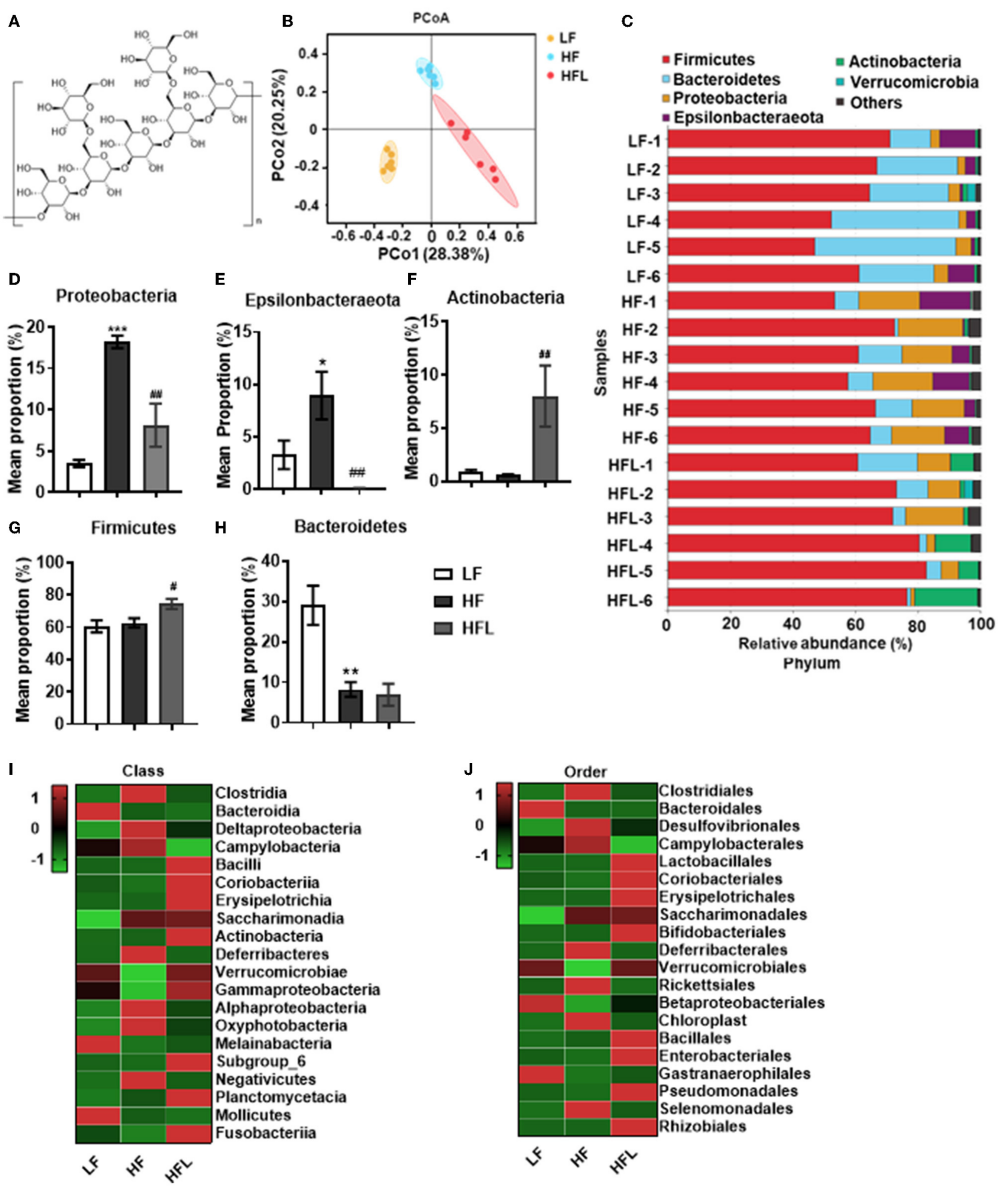
Lentinan improved HF diet-induced dysbiosis. Cecal contents microbiota composition was analyzed by 16S RNA sequencing. **(A)** The chemical structure of lentinan. **(B)** PCoA of community dissimilarity. **(C)** The taxonomic composition analysis at phylum levels among three groups. **(D–H)** Relative abundance of phylum. Proteobacteria **(D)**, Epsilonbacteraeota **(E)**, Actinobacteria **(F)**, Firmicutes **(G)**, and Bacteroidetes **(H)**. **(I,J)** The top 20 bacteria in relative abundance at the levels of class **(I)** and order **(J)** in three groups. The color of spots in the left panel of heatmap represents the Z-scores, demonstrating that all groups were represented by the Z-scores as the relative abundance levels [Z score = (actual relative abundances of a species in a specific group – mean relative abundance of the three groups)/standard deviation]. Values are means ± SEM (*n* = 6). *p < 0.05, **p < 0.01, ***p < 0.001 vs. LF mice; ^#^*p* < 0.05, ^##^*p* < 0.01 vs. HF mice.

Lentinan (Figure 1A) of purity >98% was purchased from Yuanye Biological Technology Co., Ltd (Shanghai, China). Details of sources of antibodies and other reagents used are shown in the Supplementary Data.

### Animals and Treatment

Male C57BL/6J mice aged 9 weeks were purchased from the Experimental Animal Center of Xuzhou Medical University [Xuzhou, China, SCXK (Su) 2015-0009], housed, and maintained in a 12-h light/dark photoperiod with unrestricted water and food. All animal care and experiments were carried out under protocols approved by the ethics committee of Xuzhou Medical University. After habituation to the laboratory environment for 1 week, the mice were randomly divided into four groups (*N* = 10 per group): (1) mice fed a low-fat (LF) diet (5% fat by weight) as a control (LF) group; (2) mice fed the LF diet supplemented with lentinan (500 mg/Kg in diet) as the LFL group; (3) mice receiving the HF diet (31.5% fat by weight) as the HF group; and (4) mice fed the HF diet supplemented with lentinan (500 mg/Kg in diet) as the HFL group. The detailed information of LF and HF diets is in Supplementary Table S1. The consumption of 500 mg/Kg lentinan in the diet is equivalent to dose of ~60 mg/kg body weight for mice, which was decided according to an effective dose of β-glucan at 30 mg/kg in reduction of blood glucose in rats (24) and transferred by dose translation formula between mice and rats (25). Lentinan (98%) was purchased from Yuanye Biological Technology Co., Ltd (Shanghai, China). Mice were administered the three diets for 15 weeks. Body weight and food intake were measured on the last day of each week. After 13 weeks of feeding, an intraperitoneal glucose tolerance test (GTT) was performed as detailed below. Mice were then euthanized after 15 weeks of feeding. The data of body weight, energy intake, liver weight, and GTT were collected from 10 mice per group. After the mice were sacrificed (*n* = 6 mice per group), blood was collected to detect serum LPS levels; fresh tissues of liver and intestine were collected to perform qPCR and western blot; cecum contents were collected for gut microbiota analysis. In addition, after mice were perfused with paraformaldehyde (*n* = 4 mice per group), the liver and intestinal tissues were collected for oil red O staining, Hematoxylin and eosin (H&E) staining, immunohistochemistry, and immunofluorescence.

### Glucose Tolerance Test

The GTT was performed as we have previously described (26). The detailed methods are described in the Supplementary Data.

### RNA-Sequencing Analysis

RNA sample preparation and sequencing: Total RNA was extracted from livers of mice in LF, HF, and HFL groups (three mice in each group) using TRIzol reagent kit (Invitrogen, Carlsbad, California, USA). Subsequently, the mRNA was enriched by oligo (dT) beads. The enriched mRNA was fragmented with fragmentation buffer and reverse-transcribed into cDNA with random primers. Second-strand cDNA was synthesized by DNA polymerase I, RNase H, dNTP, and buffer. The cDNA fragments were purified with a QiaQuick PCR extraction kit (Qiagen 28104), end repaired, poly(A) added, and ligated to Illumina sequencing adapters. The ligation products were size selected by agarose gel electrophoresis, PCR amplified and sequenced using Illumina HiSeq 4000 by CapitalBio Technology Co., Ltd (China).

Gene-level differential expression analyses: Differential expression genes (DEGs) analysis for mRNA was performed using R package edge R. DEGs with |log2(fold change)| value >1 and *p* < 0.05, considered as significantly modulated (27), were retained for further analysis. The KEGG pathway enrichment analysis was performed for DEGs using the Database for Annotation, Visualization, and Integrated Discovery (DAVID) (28). The protein–protein interaction (PPI) network of the proteins encoded by the DEGs was searched using the STRING database and Cytoscape software (29).

### LPS Determination

The serum concentration of LPS was determined using a chromogenic end-point TAL kit. The absorbance was measured at 545 nm using a spectrophotometer, with measurable concentrations ranging from 0.1 to 1.0 EU/ml. All samples for LPS measurements were performed in duplicate.

### Oil Red O Staining

Oil red O staining was used to examine hepatic lipid accumulation as described previously (30). The detailed methods are provided in the Supplementary Data.

### Hematoxylin and Eosin Staining

The H&E staining detection and histological assessment are described in the Supplementary Data.

### Immunohistochemistry

The immunohistochemistry methods for hepatic F4/80 and intestinal iNOS detection and quantification are provided in the Supplementary Data.

### Immunofluorescence

Jejunum segments were immunostained as described previously (31). The detailed methods are described in the Supplementary Data.

### Western Blotting

Western blot assays were performed as described previously (32). The methods are depicted in the Supplementary Data.

### Quantitative RT-PCR Analysis

Total RNA was extracted with RNA Isolator Total RNA Extraction Reagent (Vazyme, China) from the liver and jejunum, before reverse-transcription to cDNA using a high-capacity cDNA reverse transcription kit (Takara, Japan). qPCR was performed using the TransStart® Top Green qPCR SuperMix (TransGen, China) and determined on a real-time PCR detection system (Roche LightCycler480, Switzerland). The mRNA levels for specific genes were calculated using the formula 2(-ΔΔCt) and normalized by β-actin mRNA levels. All primers are listed in Supplementary Table S2.

### Gut Microbiota Analysis

DNA extraction, PCR amplification, and 16S rRNA gene sequencing: After 15 weeks of feeding, the cecum contents of mice were collected. Microbial DNA was extracted using the HiPure Stool DNA Kit (Magen, Guangzhou, China) according to the protocol recommended by the manufacturer. V3–V4 region of 16s rRNA genes was amplified by PCR with the primers 341-F, 5'-CCT ACGGGNGGCWGCAG-3' and 806-R, 5'-GGACTACHVGGG TATCTAAT-3' (33), and the amplification procedure was as follows: Initial denaturation at 94°C for 2 min, followed by denaturation at 98°C for 10 s, annealing at 65°C for 30 s, and extension at 68°C for 30 s. This round was repeated for 30 cycles, followed by a final extension at 68°C for 5 min. PCRs were performed in triplicate, and the reaction system was composed of 5 ml of 10 × KOD buffer, 5 ml of 2 mM dNTPs, 3 ml of 25 mM MgSO4, 1.5 ml of each primer (10 mM), 1 ml of KOD polymerase, and 100 ng of template DNA, with 50 ml in total. After amplification, the products were purified using the AxyPrep DNA Gel Extraction Kit (Axygen Biosciences, Union City, CA, USA) and quantified using ABI StepOnePlus Real-Time PCR System (Life Technologies, Foster City, USA). Purified products were pooled in equimolar and paired-end sequenced (PE250) on an Illumina platform according to the standard protocols.

Sequence data processing: To get high-quality clean reads, raw reads containing more than 10% of unknown nucleotides-(N) and reads with <60% of bases with a quality value (Q-value) >20 were removed using FASTP (version 0.18.0) (34). Paired-end clean reads were merged as raw tags using FLSAH (version 1.2.11) (35) with a minimum overlap of 10 bp and mismatch error rates of 2%. Noisy sequences of raw tags were filtered by QIIME (version 1.9.1) (36) pipeline under specific filtering conditions (37) to obtain high-quality clean tags. The filtering conditions were as follows: (1) break raw tags from the first low-quality base site where the number of bases in the continuous low-quality value (the default quality threshold is ≤3) reaches the set length (the default length is three); (2) then, filter tags whose continuous high-quality base length was <75% of the tag length. The clean tags were searched against the reference database (http://drive5.com/uchime/uchime_download.html) to perform reference-based chimera checking using the UCHIME algorithm. After chimeric tags were removed, the final effective tags were used for further analysis.

Bioinformatic analysis: The clean tags were clustered into operational taxonomic units (OTUs) of ≥97% similarity using UPARSE (version 9.2.64) pipeline (38). The α-diversity indices evaluating gut microbial community richness (the Ace and Chao1 estimators) and community diversity (the Shannon estimator) were calculated using Mothur (39). Principal coordinate analysis (PCoA) based on Bray–Curtis distance and permutational multivariate analysis of variance (PERMANOVA) was performed to compare the global microbiota composition after the intervention in each group at phylum, genus, and OTU levels, respectively. The difference in abundant taxa was detected by the linear discriminant analysis (LDA) effect size (LEfSe) algorithm, which emphasizes statistical significance, biological consistency, and effect relevance (40). Differences with log 10 LDA scores (absolute values) >3.0 and *p* < 0.05 were considered significant (41). The functional potential of the gut microbial communities was estimated by the PICRUSt12 algorithm (42). In univariate analysis of gut microbiota and predicted KEGG biochemical pathways in each group, a paired *t*-test or a Wilcoxon matched-pairs test was adopted and *p*-values were adjusted for multiple comparisons using the Benjamini–Hochberg false discovery rate.

### Statistical Analysis

Statistical analysis was performed using SPSS (version 20, IBM Corporation, Chicago, IL, USA). All data were tested for normality by applying the Shapiro–Wilk normality test. If normality was given, the one-way analysis of variance (ANOVA) was performed, followed by the *post-hoc*, Tukey's test for comparisons among the groups. Correlations between gut bacterial abundance and serum LPS and its binding protein and receptor in the liver and also hepatic proinflammatory and antiinflammatory cytokines were calculated using Spearman's correlation analysis. Differences were considered significant when *p* < 0.05 and marked with ^*^ or ^#^ (^*^p < 0.05, ^**^p < 0.01, ^***^p < 0.001, ^#^*p* < 0.05, ^##^*p* < 0.01, ^###^*p* < 0.001). Values are expressed as the mean ± standard error of means (SEM).

## Results

### Dietary Lentinan Supplementation Improved Gut Microbiota Dysbiosis in HF Mice

Gut microbiota dysbiosis plays an important role in NAFLD onset and progression (43, 44). Here, by 16S rRNA gene sequencing analysis, we investigated the gut microbiota after the HF diet with or without lentinan (Figure 1A) supplementation for 15 weeks. Principal coordinate analysis (PCoA) of microbiota showed a clear cluster separation among the LF, HF, and HFL groups (Figure 1B), whereas the LF diet-fed mice with lentinan supplementation had a similar cluster with the LF mice (Supplementary Figure S1A). The HF mice showed a decrease in gut microbial community richness (the Chao1 and Ace estimators) and community diversity (the Shannon estimator) (all *p* < 0.05, Supplementary Figures S1B–D), whereas lentinan prevented the reduction of gut microbial community richness (all *p* < 0.05, Supplementary Figures S1B,C). Among the LF, HF, and HFL groups, the difference in the relative abundance of microbiota at phylum was shown in Supplementary Figure S1E. Specifically, the gut microbiota of these three groups was mainly dominated by Firmicutes, Bacteroidetes, Proteobacteria, Epsilonbacteraeota, and Actinobacteria at the phylum level (Figure 1C), in which Proteobacteria and Epsilonbacteraeota increased (both *p* < 0.05, Figures 1D,E), and Bacteroidetes decreased in the HF group (*p* < 0.05, Figure 1H). Importantly, lentinan supplementation reduced the abundance of Proteobacteria and Epsilonbacteraeota and increased Actinobacteria and Firmicutes (all *p* < 0.05, Figures 1D–G and Supplementary Figure S1E). At the class level, Clostridia, Bacteroidia, Deltaproteobacteria, Campylobacteria, and Bacilli were dominant bacterial (Supplementary Figure S1F), in which Clostridia, Deltaproteobacteria, and Campylobacteria were deceased in the HF mice with lentinan supplementation compared with that of the HF group (Figure 1I). At the order level, Clostridiales, Bacteroidales, Desulfovibrionales, Campylobacterales, and Lactobacillales accounted for the majority of gut microbiota (Supplementary Figure S1G). Compared with HF mice, supplementation of lentinan decreased the relative abundance of Clostridiales, Desulfovibrionales, and Campylobacterales and increased Lactobacillales (Figure 1J).

**Figure 2:**
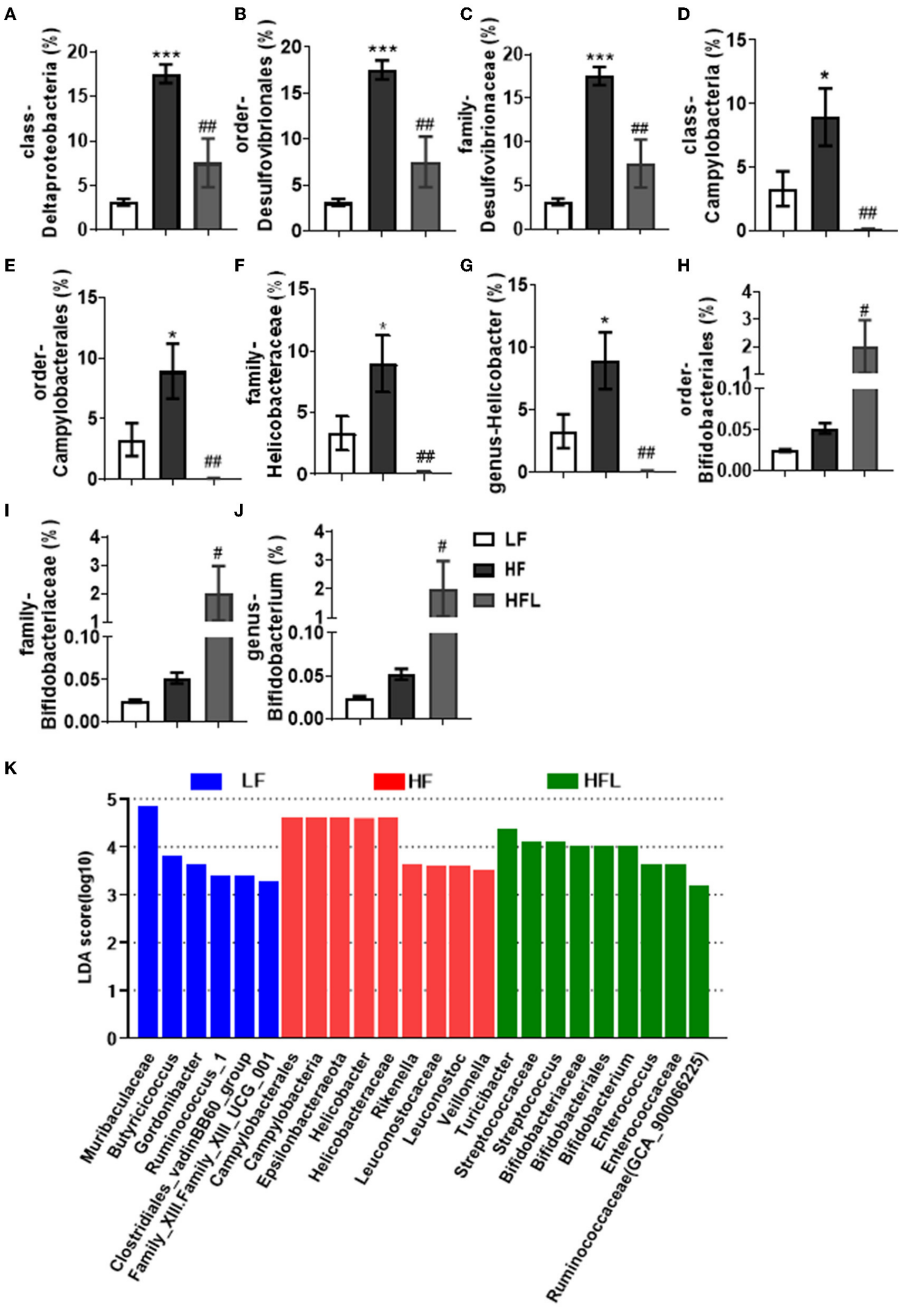
Lentinan restored the HF-induced compositional shift of gut microbial community. **(A–K)** Comparison of the representative taxonomic abundance of class Deltaproteobacteria **(A)**, order Desulfovibrionales **(B)**, family Desulfovibrionaceae **(C)**, class Campylobacteria **(D)**, order Campylobacterales **(E)**, family Helicobacteraceae **(F)**, genus *Helicobacter* **(G)**, order Bifidobacteriales **(H)**, family Bifidobacteriaceae **(I)**, and genus *Bifidobacterium* **(J)**. **(K)** LEfSe results of mice gut microbiomes. Values are means ± SEM (*n* = 6). *p < 0.05, ***p < 0.001 vs. LF mice; ^#^*p* < 0.05, ^##^*p* < 0.01 vs. HF mice.

Furthermore, in HF mice, the low taxa belonging to phylum Proteobacteria, including class Deltaproteobacteria, order Desulfovibrionales, and family Desulfovibrionaceae, were all significantly increased (Figures 2A–C and Supplementary Figures S1F–H). Phylum Epsilonbacteraeota's lower taxa, including class Campylobacteria, order Campylobacterales, family Helicobacteraceae, and genus *Helicobacter*, were significantly increased in HF mice (Figures 2D–G and Supplementary Figures S1F–I). Importantly, lentinan prevented HF-induced alterations in these microbiota belonging to the phylum Proteobacteria and Epsilonbacteraeota. Notably, bacteria belonging to Actinobacteria, order Bifidobacteriales, family Bifidobacteriaceae, and genus *Bifidobacterium* were elevated significantly in HF mice with lentinan supplementation (Figures 2H–J and Supplementary Figures S1G–I). LDA effect size (LEfSe) showed that the lower taxa bacteria of the phylum Epsilonbacteraeota, such as order Campylobacterales, class Campylobacteria, family Helicobacteraceae, and genus *Helicobacter*, belonging to phylum Proteobacteria were enriched in the HF group (Figure 2K). In HFL mice, bacteria belonging to order Bifidobacteriales, family Bifidobacteriaceae, and genus *Bifidobacterium* were elevated. In addition, genus *Turicibacter*, family Streptococcaceae, genus *Streptococcus*, genus *Enterococcus*, family Enterococcaceae, and genus *Ruminococcaceae* (GCA_900066225) were also increased in HFL mice. Moreover, KEGG functional orthologs predicted potential functional interactions between the gut microbiota and host among the LF, HF, and HFL group in 19 functional orthologs at level two (Table 1). Totally, 12 functional orthologs were significantly altered in the HF group compared with the LF group. Compared with the HF group, the lentinan supplementation was associated with microbial functional shifts in 12 functional orthologs, including carbohydrate metabolism, nucleotide metabolism, glycan biosynthesis and metabolism, metabolism of other amino acids, transcription, signal transduction, and so on.

**Table 1:** Predicted KEGG functional pathway differences at level 2 inferred from 16S rRNA gene sequence using PICRUSt after LF, HF and HFL diet.

**KO functional categories**		**LF**	**HF**	**HFL**	**LF vs. HF**	**HF vs. HFL**
**Level One**	**Level Two**				***p*-value**	***p*-value**
Metabolism	Carbohydrate Metabolism	12.062 ± 0.232	11.862 ± 0.335	12.522 ± 0.228	—	0.001
	Amino Acid Metabolism	10.842 ± 0.321	10.431 ± 0.181	10.421 ± 0.264	0.016	—
	Nucleotide Metabolism	4.562 ± 0.153	4.408 ± 0.12	4.608 ± 0.188	—	0.042
	Enzyme Families	2.569 ± 0.025	2.476 ± 0.052	2.528 ± 0.063	0.005	—
	Glycan Biosynthesis and Metabolism	2.683 ± 0.217	2.414 ± 0.151	2.108 ± 0.165	0.020	0.010
	Metabolism of Other Amino Acids	1.655 ± 0.033	1.551 ± 0.025	1.639 ± 0.075	0.003	0.008
	Biosynthesis of Other Secondary Metabolites	1.037 ± 0.044	0.947 ± 0.035	0.982 ± 0.043	0.002	—
Genetic Information Processing	Transcription	3.424 ± 0.117	3.483 ± 0.144	3.631 ± 0.077	—	0.044
Environmental Information Processing	Signal Transduction	2.139 ± 0.247	2.44 ± 0.193	2.04 ± 0.254	0.041	0.010
	Signaling Molecules and Interaction	0.194 ± 0.003	0.179 ± 0.011	0.212 ± 0.03	—	0.007
Cellular Processes	Cell Motility	3.629 ± 0.992	4.453 ± 0.691	3.155 ± 0.716	—	0.014
	Transport and Catabolism	0.415 ± 0.045	0.335 ± 0.034	0.306 ± 0.047	0.005	—
Human Diseases	Metabolic Diseases	0.106 ± 0.011	0.094 ± 0.004	0.103 ± 0.008	—	0.025
Organismal Systems	Environmental Adaptation	0.201 ± 0.011	0.221 ± 0.010	0.199 ± 0.007	0.002	0.001
	Nervous System	0.111 ± 0.004	0.103 ± 0.002	0.109 ± 0.004	0.001	0.010
	Immune System	0.105 ± 0.012	0.094 ± 0.006	0.092 ± 0.006	0.044	—
	Digestive System	0.023 ± 0.006	0.015 ± 0.003	0.014 ± 0.004	0.016	—
	Excretory System	0.02 ± 0.005	0.011 ± 0.004	0.012 ± 0.004	0.003	—
	Circulatory System	0.009 ± 0.008	0.015 ± 0.011	0 ± 0.001	—	0.007

*Values are mean (%) ± standard deviation (%). n = 6. KEGG, Kyoto Encyclopedia of Genes and Genomes; PICRUSt, Phylogenetic Investigation of Communities by Reconstruction of Unobserved States; KO, KEGG Ortholog; LF, low-fat diet; HF, high-fat diet; HFL, HF diet supplemented with lentinan*.

### Dietary Lentinan Supplementation Improved Hyperendotoxinemia, Tight Junction Proteins, and Redox Imbalance in the Jejunum of HF Mice

**Figure 3:**
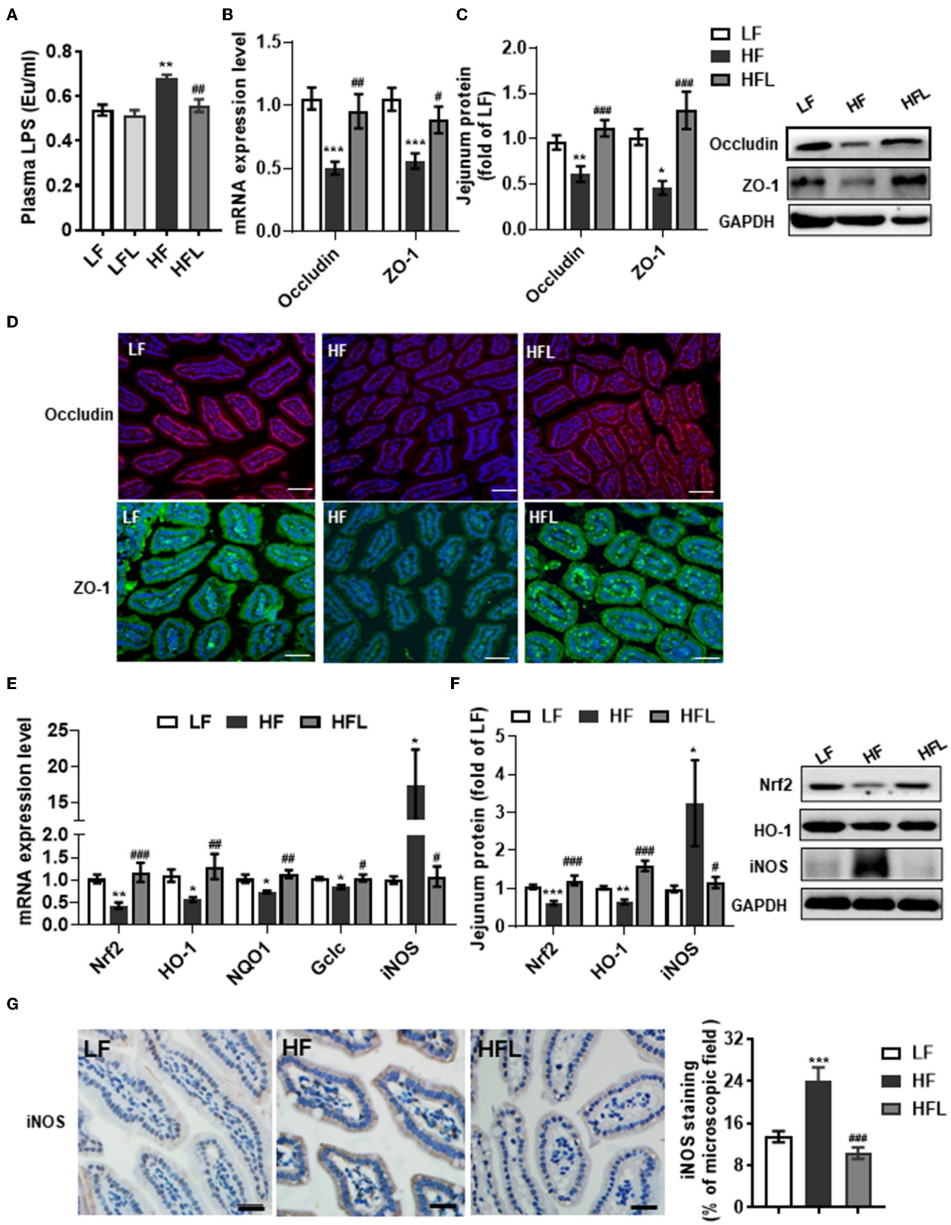
Lentinan improved intestinal barrier integrity and oxidative stress in the jejunum in HF mice. **(A)** Serum LPS levels. **(B)** The mRNA levels of occludin and ZO-1 in the jejunum. **(C)** Protein levels of occludin and ZO-1 in the jejunum. **(D)** Immunofluorescence staining of occludin and ZO-1 proteins in the jejunum sections. **(E)** The mRNA levels of Nrf2, HO-1, NQO1, Gclc, and iNOS in the jejunum. **(F)** Protein levels of Nrf2, HO-1, and iNOS in the jejunum. **(G)** iNOS protein staining of jejunum sections and quantification of iNOS protein after staining. Values are means ± SEM (*n* = 6). *p < 0.05, **p < 0.01, ***p < 0.001 vs. LF mice; ^#^*p* < 0.05, ^##^*p* < 0.01, ^###^*p* < 0.001 vs. HF mice. Scale bar: 50 μM.

Gut microbiota alteration can promote the endotoxinemia and alteration of intestinal barrier integrity (45). After identifying the capacity of lentinan to prevent gut dysbiosis, we next examined the serum LPS and the tight junction proteins, occludin and ZO-1 in the jejunum. The serum LPS level was significantly increased in the HF fed mice, whereas lentinan inhibited the elevation of serum LPS in the mice fed by HF diet, but not by LF diet (*p* < 0.01, Figure 3A). Meanwhile, the mRNA expressions of the tight junction proteins, occludin and ZO-1, were significantly lower in the jejunum of HF diet-fed mice compared with LF and HFL groups (all *p* < 0.05, Figure 3B). In line with these findings in transcription level, the protein levels of the tight junction proteins were significantly increased in the mice with lentinan supplementation (all *p* < 0.05, Figure 3C). The HF mice showed reduced immunofluorescence staining of both occludin and ZO-1, whereas clear and uniform positive staining of the tight junction proteins was found in the epithelium of jejunum in LF and HFL groups (Figure 3D), suggesting that intestinal barrier integrity was improved by lentinan supplementation.

Given that redox imbalance is the major factor for intestinal barrier dysfunction (7–10), we next investigated the effects of lentinan supplementation on antioxidants and oxidative stress markers in the jejunum. The expression of antioxidants Nrf2, HO-1, NQO1, and Gclc was decreased in the HF group, whereas lentinan supplementation significantly prevented the decrease in antioxidants induced by the HF diet (all *p* < 0.05, Figures 3E,F). Moreover, lentinan significantly inhibited the HF diet-mediated increments of iNOS protein (all *p* < 0.05, Figures 3E–G). These findings suggest lentinan supplementation attenuated redox imbalance in the intestine of HF mice.

### Dietary Lentinan Supplementation Prevented HF Diet-Induced Hepatosteatosis and Hepatic Inflammation

**Figure 4:**
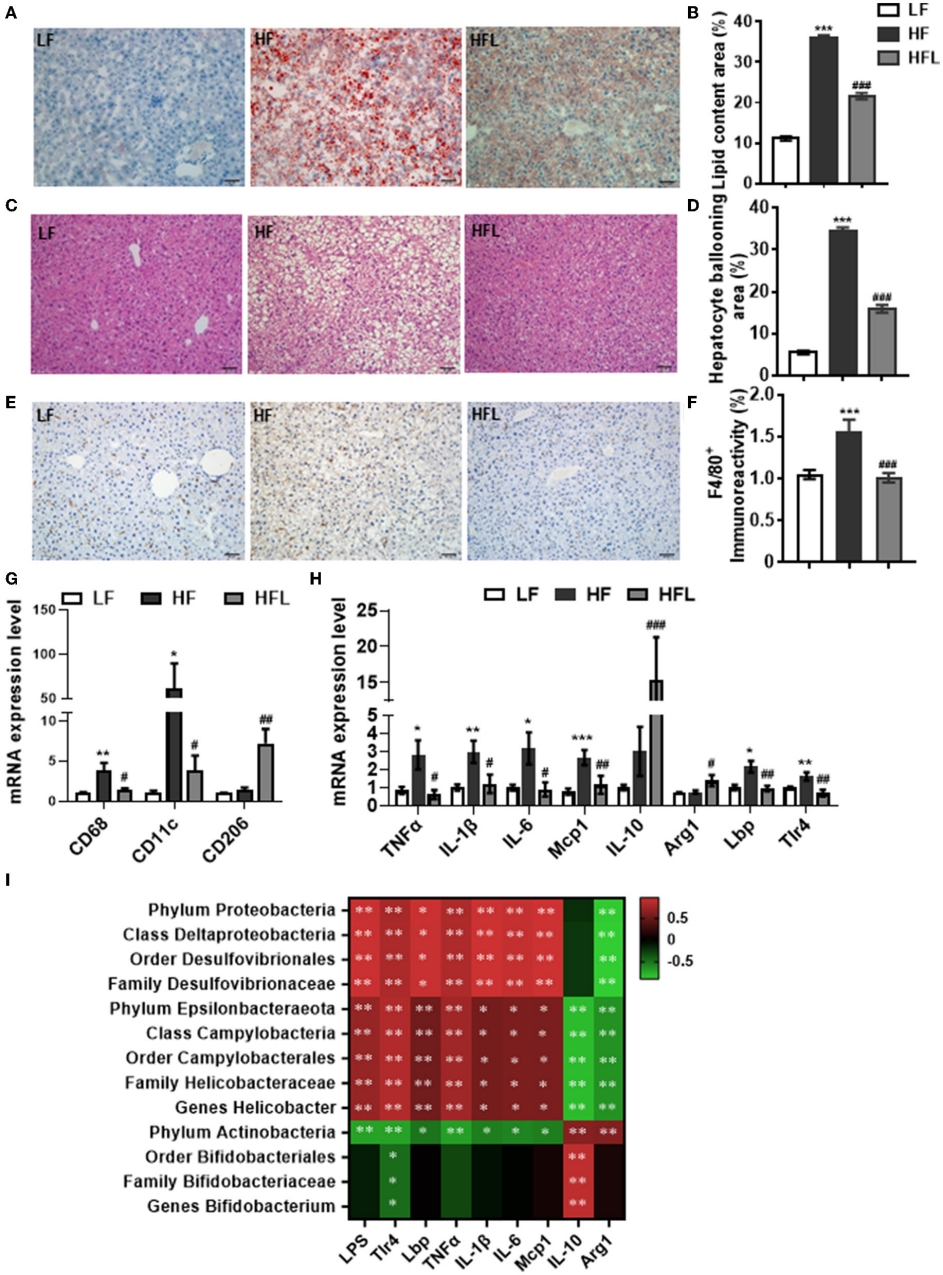
Lentinan prevented HF diet-induced hepatic lipid deposition, macrophage infiltration, and hepatic inflammation. **(A)** Oil red O staining of liver sections. **(B)** Quantification of hepatic lipid area after Oil red O staining. **(C)** H&E staining of liver sections. **(D)** Quantification of hepatic ballooning after H&E staining. **(E)** F4/80 protein staining of liver sections. **(F)** Densitometric analysis of F4/80 staining. **(G)** CD68, CD11c, and CD206 mRNA levels in liver tissues of mice. **(H)** The mRNA levels of TNFα, IL-1β, IL-6, Mcp1, IL-10, Arg1, Lbp, and Tlr4 in liver tissues. **(I)** Correlation between specific gut microbiota, serum LPS, its binding protein and receptor in the liver, and hepatic proinflammatory and antiinflammatory cytokine expression. Values are means ± SEM (*n* = 6). *p < 0.05, **p < 0.01, ***p < 0.001 vs. LF mice; ^#^*p* < 0.05, ^##^*p* < 0.01, ^###^*p* < 0.001 vs. HF mice.

Following lentinan improving gut microbiota, redox balance, and tight junction proteins, we further examined the effects of lentinan on hepatic lipid deposition and macrophage infiltration. With oil red O and H&E staining, the hepatocytes of the HF group contained larger cytoplasmic lipid droplets (*p* < 0.001, Figures 4A,B) and were enlarged with ballooning (*p* < 0.001, Figures 4C,D), compared with LF mice. These alterations in hepatic cellular morphology were significantly prevented by lentinan supplementation. Moreover, we found that the number of F4/80-positive cells (macrophage marker) was higher in HF mice compared with LF mice and HFL mice (both *p* < 0.05, Figures 4E,F), indicating that lentinan suppressed macrophage infiltration induced by HF diet. Importantly, lentinan supplementation reduced the mRNA expression of macrophage-related gene CD68 and CD11c (the marker of proinflammatory M1 type macrophages), but increased the mRNA expression of CD206 (the marker of antiinflammatory M2 type macrophages) (all *p* < 0.05, Figure 4G), suggesting that the M1 macrophage polarization was inhibited by lentinan. Furthermore, lentinan supplementation inhibited the mRNA of the proinflammatory cytokines and chemokines, TNFα, IL-1β, IL-6, and monocyte chemoattractant protein-1 (Mcp1) compared with HF mice (all *p* < 0.05, Figure 4H) and increased the expression of antiinflammatory cytokines, IL-10, and arginase I (Arg1) (both *p* < 0.05, Figure 4H). Moreover, the mRNA expressions of Lbp and Tlr4 (the LPS-binding protein and cell surface pattern recognition receptors) were significantly higher in the liver of HF mice compared to LF mice and HFL mice (*p* < 0.05, Figure 4H), which is consistent with the increase in serum LPS levels in the HF group described previously (Figure 3A).

The Spearman's correlation analysis was used to investigate the relationship between gut bacterial abundance and serum LPS and its binding protein and receptor in the liver and also hepatic proinflammatory and antiinflammatory cytokines (Figure 4I). Phylum Proteobacteria and its down taxa (class Deltaproteobacteria, order Desulfovibrionales, and family Desulfovibrionaceae), and also phylum Epsilonbacteraeota and its down taxa (class Campylobacteria, order Campylobacterales, family Helicobacteraceae and genus *Helicobacter*), were positively correlated with LPS, Lbp, Tlr4, and proinflammatory cytokines, whereas negatively correlated with antiinflammatory cytokines, IL-10, and Arg1. Phylum Actinobacteria and its down taxa (order Bifidobacteriales, family Bifidobacteriaceae, and genus *Bifidobacterium*) were negatively correlated with Tlr4 and positively correlated with IL-10. Therefore, these findings suggest that lentinan in attenuation of HF diet-induced hepatic inflammation was associated with the improvement of gut microbiota profile.

### Dietary Lentinan Supplementation Improved Glucose Intolerance and Hepatic NFκB-PTP1B-Akt-GSK3β Signaling Pathway in HF Mice

**Figure 5:**
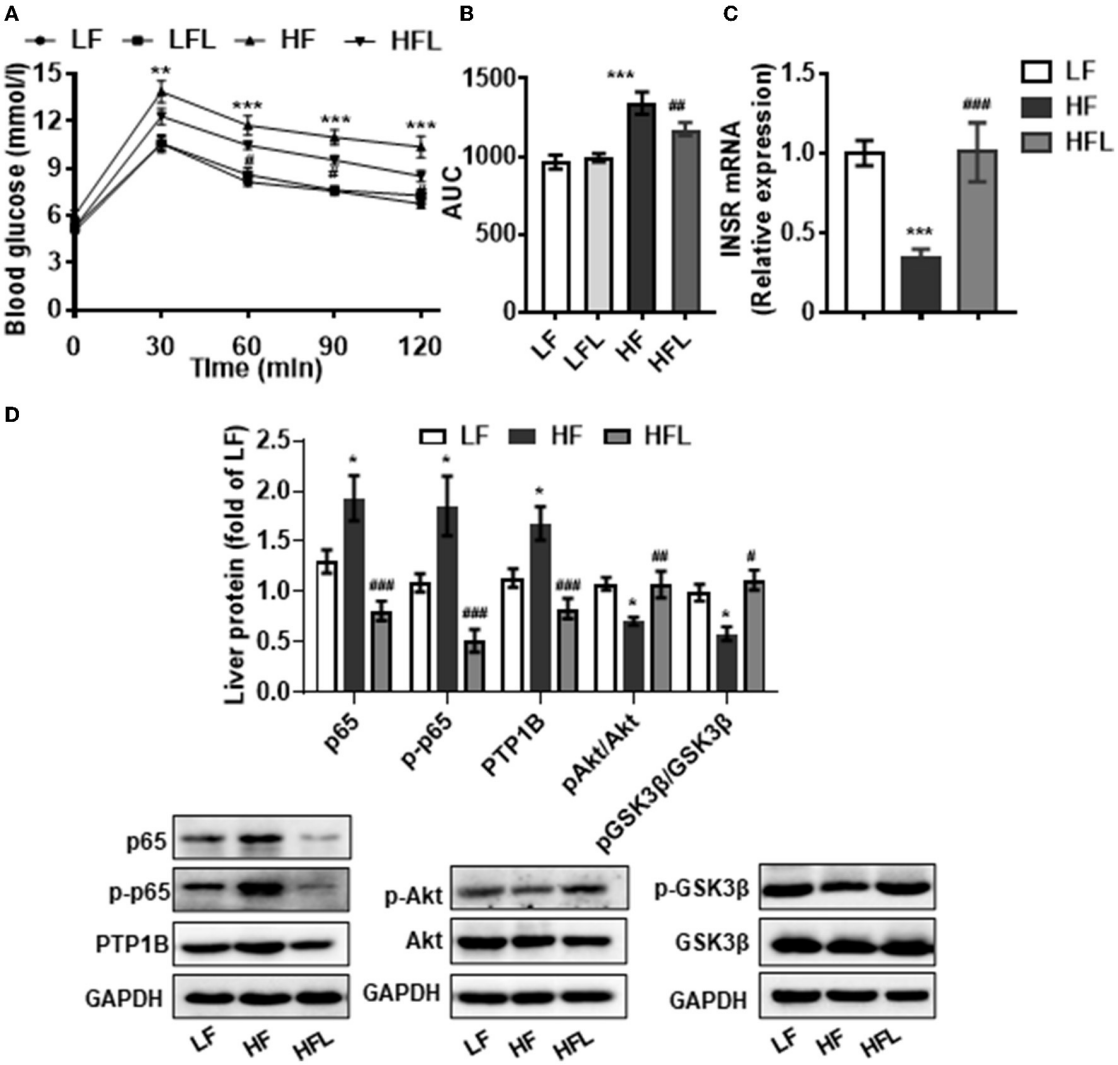
Lentinan improved glucose intolerance and NFκB-PTP1B-Akt-GSK3β signaling pathway in the liver of HF mice. **(A)** Blood glucose levels during GTT performed on week 13 and **(B)** area under the curve (AUC). **(C)** Hepatic INSR mRNA levels. **(D)** Protein levels of p65, p-p65, PTP1B, p-Akt/Akt, and p-GSK3β/GSK3β in liver tissues. Values are means ± SEM (*n* = 6). *p < 0.05, **p < 0.01, ***p < 0.001 vs. LF mice; ^#^*p* < 0.05, ^##^*p* < 0.01, ^###^*p* < 0.001 vs. HF mice.

Hepatic inflammation contributes to insulin resistance and abnormal glucose metabolism (46). Next, we determined whether lentinan could improve glucose metabolism. In the GTT test, lentinan decreased the blood glucose level at 60-, 90-, and 120-min time points (all *p* < 0.05, Figure 5A). The glucose area under curve (AUC) was markedly higher in HF mice than in LF, LFL, and HFL mice (both *p* < 0.01, Figure 5B). Consistently, mRNA expression of insulin receptor (INSR) was significantly lower in livers of HF mice than that in the other two groups (both *p* < 0.001, Figure 5C). PTP1B is a mediator of the proinflammatory (NFκB) signaling pathway in dysregulation of insulin (pAkt-pGSK3β) signaling cascade (47). We found that lentinan significantly decreased the total and phosphorylated protein levels of NFκB subunit p65 and also PTP1B protein level in livers of HF mice (all *p* < 0.001, Figure 5D). Furthermore, the phosphorylation levels of Akt and GSK3β significantly decreased in HF diet-fed mice, whereas lentinan reversed the reduction in these two insulin signaling molecules (both *p* < 0.05, Figure 5D). These results showed that lentinan improved glucose intolerance and retrieved the insulin Akt-GSK3β signaling pathway in HF mice. In addition, we observed that after the HF diet for 15 weeks, the mice had significantly higher body weight, body weight gain, energy intake, and liver weight compared with LF diet-fed mice, and lentinan supplementation prevented these changes (Supplementary Table S3). However, these metabolic indexes of LFL mice were not significantly altered compared with LF group (Supplementary Table S3).

### Dietary Lentinan Supplementation Modulated Hepatic Transcriptome Related to Metabolism and Immune Response

**Figure 6:**
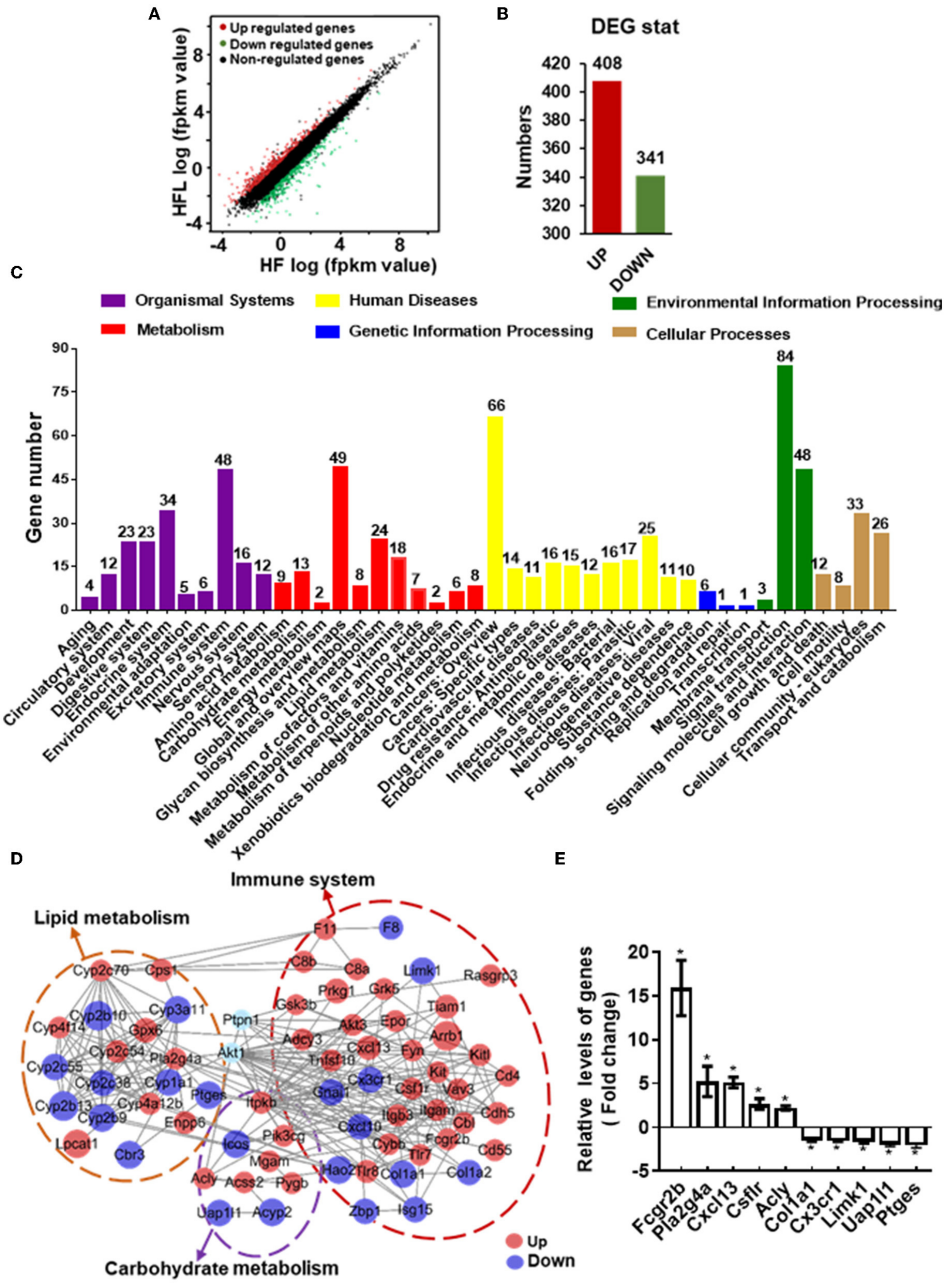
Lentinan modulated hepatic transcriptome for immune and metabolism. **(A)** Scatter plots of genes in pairwise of the HFL group vs. the HF group. The x-axis and y-axis present gene expression levels. **(B)** The number of differentially expressed genes (DEGs). **(C)** KEGG pathway classification of DEGs for the HFL group vs. the HF group. All second-level categories are grouped in top categories in different colors (*n* = 3). **(D)** PPI network by string analysis of DEGs involved in lipid metabolism, carbohydrate metabolism, immune system, and PTP1B-Akt signaling. The line indicates interaction evidence of nodes. Orange represents upregulation, and blue represents downregulation in the HFL mice by comparison with the HF mice. **(E)** qPCR analysis of the mRNA of some altered genes in the PPI network. Values are means ± SEM (*n* = 6). *p < 0.05, vs. HF mice.

To gain comprehensive profiles of lentinan supplementation on the hepatic metabolisms and inflammation at the transcriptome level, RNA-sequencing (RNA-seq) of liver tissues of HF and HFL groups was performed, followed by differentially expressed genes (DEGs) screening analysis. Consequently, lentinan supplementation modulated a total of 749 genes, including 408 upregulated genes and 341 downregulated genes (Figures 6A,B). Within the organismal system, 48 genes were significantly altered in the immune system, the most affected system (Figure 6C). In metabolism pathways, there were 146 genes altered, including 24 in lipid metabolism, 8 in glycan biosynthesis and metabolism, and 13 in carbohydrate metabolism. The top 20 KEGG pathways associated with DEGs are shown in Supplementary Table S4. Of these, the energy metabolism and immune response-related pathways were significantly affected in the liver of mice with lentinan supplementation, such as PI3K-Akt signaling pathway, metabolic pathways, arachidonic acid metabolism, and cytokine–cytokine receptor interaction. The PPI network was constructed to clarify the interaction of DEGs involved in hepatic lipid metabolism, carbohydrate metabolism, immune system, and PTP1B-Akt signaling affected by lentinan. The results demonstrated that the complex interaction network of immune and metabolism was significantly intervened by the lentinan supplementation (Figure 6D). The connectivity degree of PTP1B-Akt signaling with the PPI network was 38, indicating that PTP1B-Akt is a key node with a higher centrality value in the interaction network regulated by lentinan in HF diet-fed mice. Furthermore, we confirmed the mRNA expression of 10 DEGs by qPCR (Figure 6E).

## Discussion

In this study, we demonstrated that chronic dietary supplementation of lentinan attenuated gut microbiota alteration, intestinal tight junction deficit, and redox imbalance induced by a HF diet. In the liver, lentinan suppressed steatohepatitis and improved endotoxemia (LPS) and its downstream NFκB-PTP1B-Akt-GSK3β (inflammation–insulin) signaling pathway. Furthermore, Spearman's correlation analysis revealed that the gut phylum Proteobacteria, Epsilonbacteraeota, and Actinobacteria, and also their next level taxa, were correlated with LPS-related-binding protein and receptor, and inflammatory cytokines in the liver. Previous animal and epidemiological studies suggest that mushroom intake has beneficial effects for steatohepatitis and NALFD (48–50). For example, in a large population-based study (a cross-sectional study of 24,236 adults), a high intake of mushrooms is associated with a low prevalence of NAFLD (50). However, the detailed mechanisms of mushroom in improving NAFLD have not been investigated. Here, importantly, our findings of lentinan (the main ingredient of mushroom) in benefits to the gut–liver axis suggest that lentinan as a prebiotic contributes to mushroom consumption in preventing western-style diets induced NAFLD.

Previous clinical studies have reported that the richness and diversity of gut microbiota are decreased in patients with NAFLD (51, 52). Furthermore, at the phylum level, the proportion of Bacteroidetes is decreased in patients with NAFLD (53). The progression of NAFLD is correlated with an increase in Proteobacteria (52). In this study, in the HF diet-induced NAFLD mouse model, we found that both the richness and the diversity were decreased in the gut microbiota. At phylum levels, the abundance of Bacteroidetes was decreased, whereas the Proteobacteria was increased in HF diet-fed mice. Therefore, the findings of our mouse study further suggest that overconsumption of dietary fat contributes to gut microbiota dysbiosis (in richness, diversity, and composition) in the NAFLD. Importantly, in this study, supplementation of lentinan prevented HF diet-induced gut microbiota alteration in richness and composition. At phylum levels, lentinan ameliorated the elevation of the Proteobacteria and Epsilonbacteraeota induced by a HF diet. Moreover, lentinan increased the abundance of Actinobacteria and Firmicutes at phylum levels. With LDA analysis, lentinan supplementation promoted bacteria that belongs to the phylum Actinobacteria, including order Bifidobacteriales, family Bifidobacteriaceae, and genus *Bifidobacterium*. These bacteria have been reported to be probiotics to improve lipid metabolism and inhibit the progression of NAFLD (54, 55). Therefore, lentinan in improving the gut microbiota richness and composition in the above probiotics may contribute to the prevention of NAFLD induced by the HF diet.

In this study, lentinan supplementation promoted the probiotics for gut health, such as genus *Bifidobacterium*. It is reported that microbiomes belonging to the genus *Bifidobacterium* are associated with decreased intestinal permeability of infants (56), and the administration of *Bifidobacterium infantis* increases the tight junction proteins, occludin and claudin four, in the intestine of a neonatal mouse model of necrotizing enterocolitis (57). The probiotics in *Bifidobacterium* improving gut integrity have been considered due to their potentials in reducing oxidative stress. For example, *Bifidobacterium lactis HN019* supplemented in milk reduces nitric oxide metabolites levels in patients with metabolic syndrome (58). A yogurt enriched with *Bifidobacterium lactis* decreases malondialdehyde (a prooxidant biomarker) in patients with diabetes mellitus type two (59). Importantly, we found that lentinan supplementation significantly attenuated the alterations in oxidative stress marker iNOS and antioxidants Nrf2, HO-1, NQO1, and Gclc in the small intestinal tissue induced by the chronic HF diet. Overall, these findings suggest that lentinan supplementation in promoting probiotics, such as genus *Bifidobacterium*, contributes to attenuating cellular redox imbalance and intestinal barrier disruption.

In this study, we found a dramatic reduction in tight junction proteins (occludin and ZO-1) in the jejunum of HF mice. It is reported that the intensity of ZO-1 staining is significantly lower in the duodenum of patients with NAFLD with increased intestinal permeability (60). The abnormality of intestine permeability is related to the increased bacterial overgrowth prevalence in the small intestine of these patients (60). Interestingly, in this study, we revealed that the bacteria of phylum Proteobacteria and Epsilonbacteraeota were significantly increased in the small intestine of NAFLD mice induced by the HF diet. Proteobacteria is a major source of translocated antigen LPS from the gut (61, 62). It is reported that LPS exposure causes oxidative stress in the gut (63). NO/iNOS system mediates LPS-induced barrier dysfunction in the intestine, evidenced by that the administration of NOS inhibitor attenuates LPS-induced tight junction disruption in the ileum and colon of mice (9). Therefore, the increased Proteobacteria and its derived LPS may contribute to gut barrier dysfunction and redox imbalance. In addition, some bacteria of phylum Epsilonbacteraeota, such as order Campylobacterales, genus *Campylobacter*, and genus *Helicobacter*, are tolerant of bile acids and promote hepatic inflammation (64). Flagellins and other 19 proteins of *Campylobacter jejuni* are associated with the bile adaptation (65). The intragastrical inoculation of *Helicobacter hepaticus* induces chronic hepatitis and fibrosis in mice with the activation of p65 in the liver (66). Therefore, the above findings suggest that the bile resistance property of bacteria in phylum Epsilonbacteraeota may allow them to colonize the bile and liver to promote chronic hepatitis in the NAFLD mice induced by the HF diet. Importantly, we found that lentinan supplementation improved tight junction proteins (occludin and ZO-1) deficits in the small intestine and concurrently decreased the proportion of phylum Proteobacteria and Epsilonbacteraeota and serum LPS in HF diet-fed mice. Therefore, lentinan in the prevention of the dysbiosis of Proteobacteria, overtranslocation of its derived metabolite LPS into blood circulation and the inhibition of bile-resistant Epsilonbacteraeota may be involved in the improvement of gut barrier dysfunction and hepatic inflammation induced by chronic HF diet.

The liver is an important immunological organ, in which the immune system is activated after exposure to gut microbiome-derived factors, such as LPS (13, 67). We found that LPS-binding protein and cell surface pattern recognition receptor, Lbp and Tlr4, were significantly increased in the liver of mice fed the HF diet. Consistently, in a clinical study, Tlr4 mRNA expression is upregulated in hepatic biopsy tissue of patients with NASH with increased serum LPS (17). Tlr4 triggers the initial activation of the NFκB signaling pathway, which is necessary for the transcription of proinflammatory cytokines (TNFα, IL-6, and IL-1β) and overexpression of PTP1B (the inhibitor of insulin signaling) (15, 68). In this study, we found that the HF diet promoted steatohepatitis and glucose intolerance in mice, which had M1 proinflammatory macrophage polarization, elevated NFκB and PTP1B level and impaired insulin Akt-GSK3β signaling pathway in the liver. Importantly, supplementation with lentinan attenuated these adverse effects induced by the HF diet. Furthermore, the RNA-seq profile of the liver transcriptome followed by the KEGG pathways analysis also confirmed that the PI3K-Akt signaling pathway and cytokine–cytokine receptor interaction in the liver were significantly affected by lentinan. Consistently, the PPI network analysis showed that PTP1B and Akt1 are vital nodes in the network among metabolism and the immune system. In addition, the KEGG pathway analysis suggests that lentinan significantly modulated the arachidonic acid metabolism pathway, which is reported to be involved in the development and progression of NAFLD (69). However, the detailed mechanism of the arachidonic acid pathway involving lentinan improving NAFLD requires further investigation. Furthermore, according to the current mice study, supplementation of 368 mg lentinan per day may attenuate the NAFLD in humans. However, clinical trials would be required to determine the optimum dose in future human studies.

## Conclusion

**Figure 7:**
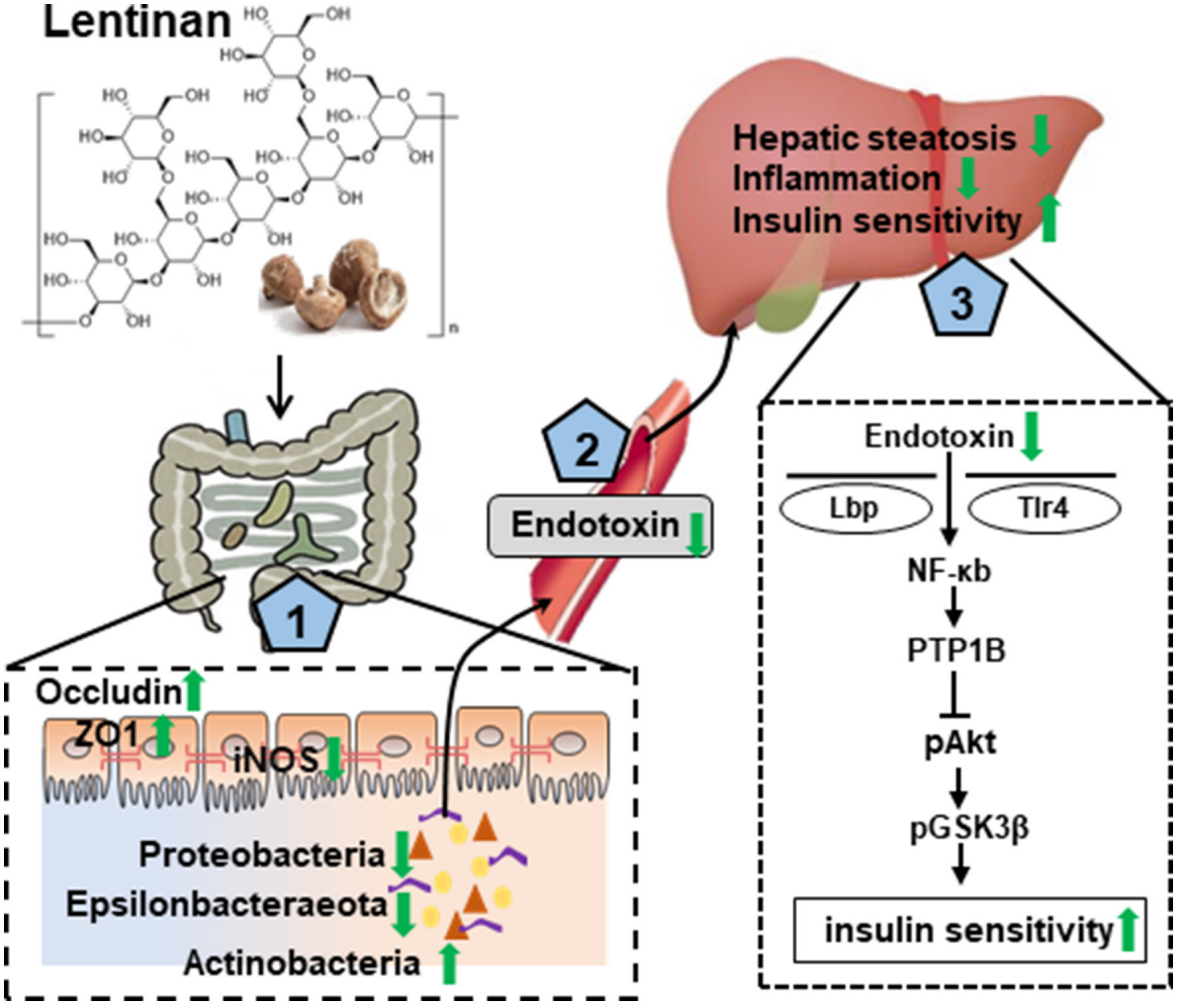
Graphical summary of the protective effect of lentinan on the development of NAFLD *via* the gut–liver axis. Lentinan showed conservation of appropriate intestinal microbiota composition, resetting cellular redox balance (activation of antioxidants and inhibition of iNOS), and increasing tight junction proteins for gut epithelial integrity (1), which contribute to reduce endotoxin translocation into circulation and liver (2) and thereby to mitigate the adverse consequences of HF consumption, that is, hepatic steatosis and inflammation, and insulin resistance (3). Green arrows represent lentinan's effects.

In summary, this study with the NAFLD mouse model induced by the HF diet, chronic lentinan supplementation preserved the conservation of gut microbiota in richness and appropriate composition. Remarkably, lentinan supplementation promoted genus *Bifidobacterium* (important for intestinal barrier integrity and redox balance) and inhibited the proliferation and growth of bacteria belonging to the phylum Proteobacteria (a major source of translocated antigen LPS) and bile-resistant Epsilonbacteraeota. Lentinan supplementation enhanced tight junction proteins and reset cellular redox balance (activation of antioxidants and inhibition of iNOS) in the small intestine and also improved steatohepatitis and NFκB-PTP1B-Akt-GSK3β (inflammation–insulin) signaling pathway in the liver. In addition to highlight the mechanisms underlying lentinan in improving NAFLD through the gut–liver axis (Figure 7), the findings of this study suggest increasing intake of mushroom ingredient, lentinan, could be a plausible strategy to mitigate the adverse effects of western-style diets on the gut microbiota and gut–liver axis in NAFLD.

## Data Availability Statement

RNA-Seq analysis raw reads were deposited into the NCBI Sequence Read Archive (SRA) database, accession number PRJNA784639. All 16S rRNA raw data were submitted to the NCBI Sequence Read Archive (SRA) database with the accession number PRJNA784743.

## Ethics Statement

The animal study was reviewed and approved by the Ethics Committee of Xuzhou Medical University.

## Author Contributions

XY and YY: conceptualization and funding acquisition. MZhe: methodology and data curation. XG, MZho, LZ, XL, ML, and HL: validation. MZhe, JZ, and NP: formal analysis. XY: investigation. XY and MZhe: writing—original draft preparation. YY and KZ: writing, reviewing, and editing. X-FH and YY: visualization. All authors contributed to the article and approved the submitted version.

## Funding

The study was funded by the National Natural Science Foundation of China (81870854, 82071184, and 81800718), the Natural Science Foundation of the Jiangsu Higher Education Institutions of China (18KJB310015 and 19KJA560003), the Natural Science Foundation of Jiangsu Province (No. BK20211055), the Jiangsu Shuangchuang Program, the Priority Academic Program Development of Jiangsu Higher Education Institutions (PAPD) in 2014, the Starting Foundation for Talents of Xuzhou Medical University (D2018006 and D2018003), and the Jiangsu Graduate Innovation Program (No. KYCX20_2469 and No. KYCX21_2637).

## Conflict of Interest

The authors declare that the research was conducted in the absence of any commercial or financial relationships that could be construed as a potential conflict of interest.

## Publisher's Note

All claims expressed in this article are solely those of the authors and do not necessarily represent those of their affiliated organizations, or those of the publisher, the editors and the reviewers. Any product that may be evaluated in this article, or claim that may be made by its manufacturer, is not guaranteed or endorsed by the publisher.

## Abbreviations

AUC: area under curve
DEGs: differentially expressed genes
Gclc: glutamate-cysteine ligase catalytic subunit
GTT: glucose tolerance test
HF: high-fat diet
HFL: high-fat diet supplemented with lentinan
HO-1: haem oxygenase 1
iNOS: inducible NO synthase
INSR: insulin receptor
Lbp: lipopolysaccharide-binding protein
LF: low-fat diet
LPS: lipopolysaccharide
Mcp1: monocyte chemoattractant protein-1
NAFLD: nonalcoholic fatty liver
NFκB: nuclear factor kappa B
NQO1: NAD(P)H quinone dehydrogenase 1
Nrf2: nuclear factor-E2-related factor 2
PTP1B: protein tyrosine phosphatase 1B
ROS: reactive oxygen species
Tlr4: toll-like receptor 4
TNFα: tumor necrosis factor alpha
ZO-1: zonula occludens-1.
