# Supplementary material for: Lentinan Supplementation Protects the Gut–Liver Axis and Prevents Steatohepatitis: The Role of Gut Microbiota Involved
Source: Front Nutr. 2022 Jan 20;8:803691. doi: 10.3389/fnut.2021.803691 (PMC8810540; doi:10.3389/fnut.2021.803691)
Supplement: Supplementary file 1 [file Presentation_1.PDF]

Supplementary Materials

Materials and Methods

Materials

Lentinan was purchased from Yuanye Biological Technology Co., Ltd (Shanghai, China). A chromogenic end-point TAL kit for LPS measurement was obtained from Xiamen Bioendo Technology Co., Ltd (Xiamen, China) and a HiPure stool DNA kit from Magen (Beijing, China). Antibodies against Occludin and PTP1B were purchased from Abcam (UK). Antibodies against Occludin, F4/80 and iNOS, Alexa Fluor® 488-conjugated Goat Anti-Rabbit IgG (H+L), HRP conjugated Goat Anti-Rabbit IgG (H+L), and DAB Immunohistochemistry Color Development Kit were purchased from Wuhan Servicebio Technology Co., Ltd (Wuhan, China). Antibodies against Nrf2, and iNOS were provided by Shenyang Wanlei Biological Technology Co., Ltd (Shenyang, China). Antibody against ZO-1 was purchased from Wuhan Sanying Biology Technology Co., Ltd (Wuhan, China). Antibodies against NF-κB p65 and phospho-NF-κB p65 (AN371) were purchased from Beyotime Biotechnology Co., Ltd (Shanghai, China). Antibodies against Akt, p-Akt (Ser473), Gsk3βand p-Gsk3β (Ser9) were provided by Cell Signaling Technology Co., Ltd (Boston, USA). Antibody against GAPDH was provided by ABclonal Biotechnology Co., Ltd (China).

GTT

Mice were intraperitoneally injected with glucose (2g/kg) after fasting overnight. Blood glucose levels in the tail-tip blood were measured using a glucose monitor at 0, 30, 60, 90 and 120 minutes following the glucose injection. The area under the curve (AUC) was calculated by the summation of trapezoids.

Oil Red O staining

Briefly, frozen liver sections (10 μm) were stained with 0.5% Oil Red O (Sigma-Aldrich) for 15 minutes and then washed. Three fields from three sections of each mouse were viewed under a Leica microscope, and digital photographs were captured. Image J software was used to quantify the staining, which corresponds to the percentage of stained lipid droplets on an area of each slide.

Haematoxylin and eosin (H&E) staining

Briefly, the liver tissues were cut and fixed with 4% paraformaldehyde and were embedded in paraffin. Liver sections (5 μm) were stained with hematoxylin and eosin for 30s each to determine

the degree of liver damage. Three fields from three sections of each mouse were viewed under a Leica microscope, and digital photographs were captured. The histological parameters of steatosis and ballooning were scored.

Immunohistochemistry

The liver and jejunum were removed and fixed overnight in 4% (w/v) neutralized paraformaldehyde solution. Samples were subsequently washed twice in phosphate buffer saline solution, dehydrated, and then embedded in paraffin for histological analysis. Sections (5 μm thickness) were obtained from paraffin blocks and placed on glass slides. Slides were incubated overnight at 4 °C with anti-F4/80 (1: 2000 dilution) for liver tissue and anti-iNOS (1: 500 dilution) for jejunum tissue diluted in blocking buffer. Three fields per section from three sections of each mouse were viewed under a Leica microscope, and digital photographs were captured. Image J software was used to quantify the area of F4/80 and iNOS immunoreactivity on each slide.

Immunofluorescence

Briefly, the jejunum tissues were cut into 5 μm sections, deparaffinized in xylene, and rehydrated in decreasing concentrations of ethanol. Ethylenediaminetetraacetic acid (PH 8.0) was used for antigen retrieval. To avoid nonspecific binding, the sections were preincubated in 1% bovine serum albumin in phosphate-buffered saline for 30 min. Rabbit polyclonal anti-occludin (1:3000; Servicebio) and rabbit polyclonal anti-ZO-1 (1:200; Servicebio) were used as the primary antibodies and were incubated on the sections for 20 h at 4 ℃. Alexa dye-conjugated secondary antibodies (1:300, Alexa flour® 488 and HRP-labeled Goat Anti-Rabbit IgG (H+L), Servicebio) were applied at room temperature for 50 min. Counterstaining with 4, 6-diamidino-2-phenylindole was used to visualize the nuclei (Servicebio). Finally, all sections were imaged under the microscope (OLYMPUS IX51) and digital photographs were captured.

Western blotting

Proteins were extracted from the liver and jejunum in cell lysis buffer (containing RIPA, Protease Inhibitor Cocktail and 1 mM PMSF). After quantification by BCA assay, 40-80 μg of protein was separated using 10% SDS-PAGE and then electrotransferred to polyvinylidene difluoride (PVDF) membrane. Western blot assays were performed using primary antibodies specific for NF-κB p65, p-NF-κB p65 (Ser536), PTP1B, Akt, p-Akt (Ser473), Gsk3β, p-Gsk3β

(Ser9), Occludin, ZO-1, Nrf2, HO-1, iNOS and GAPDH. Immunodetection was performed using Clarity™ ECL western blot substrate (Bio-Rad, USA) and visualized with the ChemiDoc Touch imaging system (Bio-Rad, USA). All quantitative analyses for total and cytosolic proteins were normalized to GAPDH.

Table S1 Composition of the diets.

	LF	HF
Energy (%)		
Fat	12	55
Carbohydrate	67	30
Protein	21	15
Component, (g/100g)		
Carbohydrate from cornstarch, sucrose (g)	63.0	38.5
Fat from sunflower, lard (g)	5.0	31.5
Protein from casein, gelatine (g)	19.3	18.3
Total fibre/100g food (g)	5.1	5.1
cellulose (g)	5.1	5.1

LF was refined purified diet; LFLwas a diet mixed 500 mg/kg lentinan into LF diet . HF was a high-fat (315 g/kg from fat, 31.5% by weight) and fiber-deficient (50g/kg from cellulose, 5% by weight) diet. HFL was a diet of 500 mg/kg lentinan into the HF diet. Foods were made from semi-synthetic materials according to the recommendation of “AIN93 Diet for Laboratory Rodents”.

Table S2. Primers used for PCR

Mouse Gene	Forward Primer (5'→3')	Reverse Primer (5'→3')
CD68	TCACCTTGACCTGCTCTCTCTAA	GCTGGTAGGTTGATTGTCGTCTG
CD11C	CTGGATAGCCTTTCTTCTGCTG	GCACACTGTGTCCGAACTCA
CD206	ATCCTGGTGGAAGAAGAAGTAGCCT	GAGTAGTGGTTGGAGAAACAGGCAG
TNFα	CTTGTTGCCTCCTCTTTTGCTTA	CTTTATTTCTCTCAATGACCCGTAG
IL-1β	TGGGAAACAACAGTGGTCAGG	CTGCTCATTCACGAAAAGGGA
IL-6	TCACAGAAGGAGTGGCTAAGGACC	ACGCACTAGGTTTGCCGAGTAGAT
IL-10	GGAAGACAATAACTGCACCCACT	CAACCCAAGTAACCCTTAAAGTCC
Mcp1	GTGCTGACCCCAAGAAGGAATG	TGAGGTGGTTGTGGAAAAGGTAGTG
Arg1	GGCAACCTGTGTCCTTTCTCCT	CCCAGCTTGTCTACTTCAGTCATG
Lbp	TGGCACCCAAGTATAAGAA	ATCCAGACAAGGCACAAG
Tlr4	TTCACCTCTGCCTTCACTAC	GACACTACCACAATAACCTTCC
Occludin	CTATGGGACAGGGCTCTTTGGA	AGGAAGCGATGAAGCAGAAGGC
Zo1	AATGAGGATGAGGTTGTGTC	TTGTAGTTGTGAAGAGATGGTG
iNOS	CTTGGAGCGAGTTGTGGATTGTC	TAGGTGAGGGCTTGGCTGAGTG
Nrf2	CTTTAGTCAGCGACAGAAGGAC	AGGCATCTTGTTTGGGAATGTG
Ho1	GATAGAGCGCAACAAGCAGAA	CAGTGAGGCCCATACCAGAAG
Nqo1	AGGATGGGAGGTACTCGAATC	TGCTAGAGATGACTCGGAAGG
Gclc	CTACCACGCAGTCAAGGACC	CCTCCATTCAGTAACAACTGGAC
Cxcl13	GGCCACGGTATTCTGGAAGC	GGGCGTAACTTGAATCCGATCTA
Fcgr2b	AGGGCCTCCATCTGGACTG	GTGGTTCTGGTAATCATGCTCTG
Csflr	TGTCATCGAGCCTAGTGGC	CGGGAGATTCAGGGTCCAAG
Pla2g4a	CAGCACATTATAGTGGAACACCA	AGTGTCCAGCATATCGCCAAA
Acly	CAGCCAAGGCAATTTCAGAGC	CTCGACGTTTGATTAACTGGTCT
Cx3cr1	GAGTATGACGATTCTGCTGAGG	CAGACCGAACGTGAAGACGAG
Ptges	GGATGCGCTGAAACGTGGA	CAGGAATGAGTACACGAAGCC
Uap1l1	CATCTCCTGCGCTTCTATGCC	CGTGTCTCTTGGTCACAGC
Col1a1	GCTCCTCTTAGGGGCCACT	CCACGTCTCACCATTGGGG
Limk1	ATGAGGTTGACGCTACTTTGTTG	CTACACTCGCAGCACCTGAA
β-actin	TGAGAGGGAAATCGTGCGTGAC	GCTCGTTGCCAATAGTGATGACC

Table S3. The metabolic parameters of LF, LFL, HF and HFL mice after 15 weeks of intervention.

Metabolic parameters	LF	LFL	HF	HFL
Body weight (g)	28.34±1.51	28.76±1.32	43.12±3.52***	31.87±4.05###
Body weight gain (g)	3.99±1.86	3.62±2.26	16.41±3.76***	4.52±3.59###
Energy intake (kcal/day/mouse)	11.52±0.85	10.96±0.59	14.19±0.88***	13.67±0.06
Liver weight (g)	1.52±0.10	1.51±0.29	2.53±0.74**	1.62±0.22##
Liver weight / Body weight (%)	4.52±0.23	4.50±0.77	5.16±0.97	3.86±0.43#

Values are means ± standard deviation. LF: low-fat diet; LFL: low-fat diet supplemented with lentinan. HF: high-fat diet; HFL: high fat diet supplemented with lentinan. $^{**}p < 0.01$, $^{***}p < 0.001$ versus LF mice. $^{\#}p < 0.05$, $^{\#\#}p < 0.01$, $^{\#\#\#}p < 0.001$ versus HF mice (n=6 per group).

Table S4. The top 20 KEGG Pathways and the associated genes in the liver are significantly affected by lentinan.

Term	Input number	Background number	*p* Value	Corrected *p* Value
Rap1 signaling pathway	22	214	1.27E-08	3.74E-06
Chemical carcinogenesis	15	93	1.35E-08	3.74E-06
Arachidonic acid metabolism	13	89	3.48E-07	3.75E-05
Focal adhesion	19	203	4.72E-07	4.61E-05
Proteoglycans in cancer	19	205	5.41E-07	4.85E-05
Ras signaling pathway	20	229	6.51E-07	5.39E-05
Retinol metabolism	12	88	1.89E-06	0.000135
PI3K-Akt signaling pathway	24	343	2.1E-06	0.000142
Cytokine-cytokine receptor interaction	20	264	4.93E-06	0.000295
Amoebiasis	12	109	1.39E-05	0.000747
Prion diseases	7	34	2.66E-05	0.001364
ECM-receptor interaction	10	83	3.57E-05	0.001602
Platinum drug resistance	9	77	0.000108	0.004151
Metabolism of xenobiotics by cytochrome P450	8	65	0.000188	0.00674
Hematopoietic cell lineage	9	85	0.000214	0.007093
Steroid hormone biosynthesis	9	86	0.000232	0.007349
Metabolic pathways	49	1278	0.000243	0.007466
Osteoclast differentiation	11	129	0.000258	0.007719
Axon guidance	13	176	0.000276	0.007817
Pathways in cancer	21	394	0.000348	0.00937

Figure S1

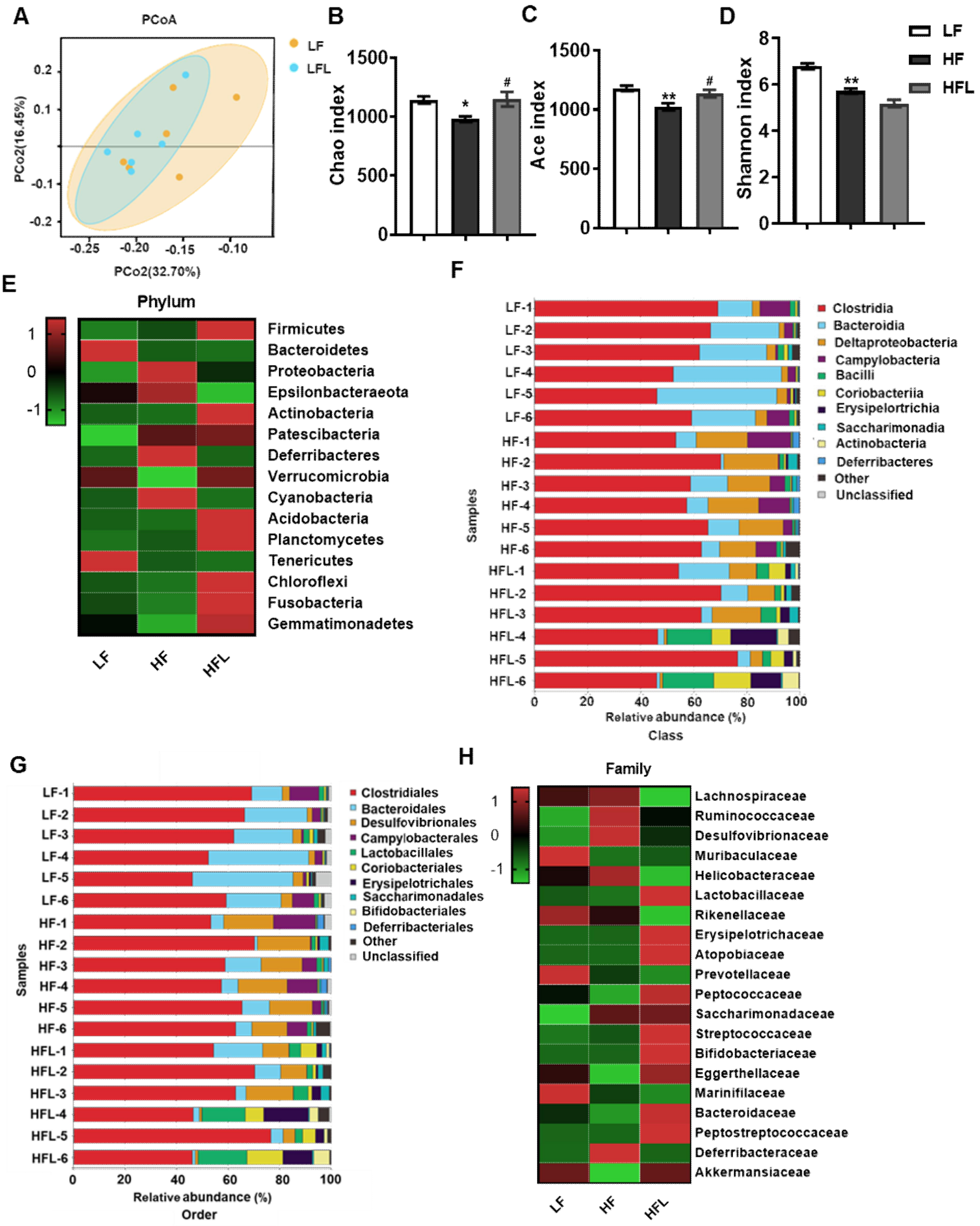

A
PCoA
LF
LFL
PCo2(16.45%)
PCo2(32.70%)
B
Chao index
C
Ace index
D
Shannon index
LF
HF
HFL
E
Phylum
Firmicutes
Bacteroidetes
Proteobacteria
Epsilonbacteraeota
Actinobacteria
Patescibacteria
Deferribacteres
Verrucomicrobia
Cyanobacteria
Acidobacteria
Planctomycetes
Tenericutes
Chloroflexi
Fusobacteria
Gemmatimonadetes
F
Samples
Relative abundance (%)
Class
Clostridia
Bacteroidia
Deltaproteobacteria
Campylobacteria
Bacilli
Coriobacteriia
Erysipelortrichia
Saccharimonadia
Actinobacteria
Deferribacteres
Other
Unclassified
G
Samples
Relative abundance (%)
Order
Clostridiales
Bacteroidales
Desulfovibrionales
Campylobacterales
Lactobacillales
Coriobacteriales
Erysipelotrichales
Saccharimonadales
Bifidobacteriales
Deferribacteriales
Other
Unclassified
H
Family
Lachnospiraceae
Ruminococcaceae
Desulfovibrionaceae
Muribaculaceae
Helicobacteraceae
Lactobacillaceae
Rikenellaceae
Erysipelotrichaceae
Atopobiaceae
Prevotellaceae
Peptococcaceae
Saccharimonadaceae
Streptococcaceae
Bifidobacteriaceae
Eggerthellaceae
Marinifilaceae
Bacteroidaceae
Peptostreptococcaceae
Deferribacteraceae
Akkermansiaceae

I

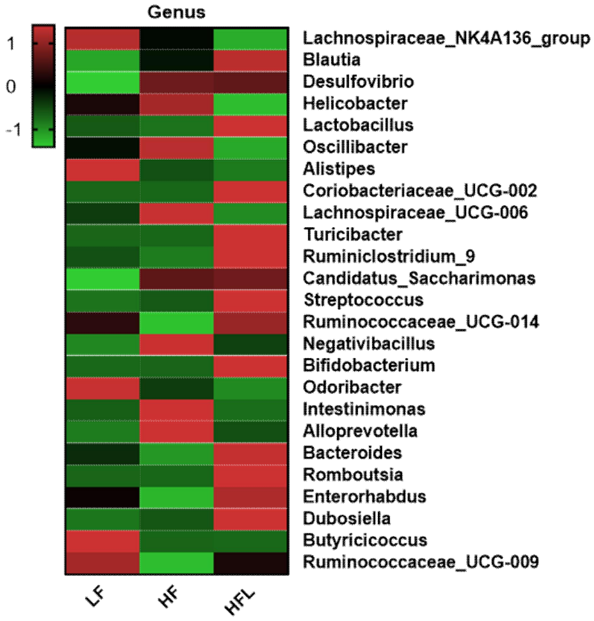

Figure S1 Lentinan supplementation altered the gut microbiota in LF, HF and HFL group mice. (A) Principal coordinates analysis of community dissimilarity. (B) Chao index. (C) Ace index. (D) Shannon index. (E) The heatmap of relative abundance of different bacteria at the phylum. (F-G) The Taxonomic composition analysis on Class (F) and order (G) levels of three group mice**.** The heatmap of relative abundance of different bacteria the top 20 at the family (H), as well as the top 25 at the levels of genus (I) in three groups. The color of spots in the left panel represents the Z-scores, demonstrating all groups were represented by the Z-scores as the relative abundance levels (Z score = [actual relative abundances of a species in a specific group − mean relative abundance of the the three group]/standard deviation). $*p < 0.05$, $**p < 0.01$, $***p < 0.001$ versus LF mice; $^{\#}p < 0.05$, $^{\#\#}p < 0.01$, $^{\#\#\#}p < 0.001$ versus HF mice.
